# Thermoregulated Coacervation, Metal-Encapsulation and Nanoparticle Synthesis in Novel Triazine Dendrimers

**DOI:** 10.3390/molecules21050599

**Published:** 2016-05-11

**Authors:** Fermín Ramírez-Crescencio, Alan E. Enciso, Mirza Hasan, Viviana C. P. da Costa, Onofrio Annunziata, Rocío Redón, Jeffery L. Coffer, Eric E. Simanek

**Affiliations:** 1 Centro de Ciencias Aplicadas y Desarrollo Tecnológico, Universidad Nacional Autónoma de México, Cd. Universitaria, A.P. 70–186, C.P., Cd. Mx. 04510, Mexico; qfermin@gmail.com (F.R.-C.); rredon@unam.mx (R.R.); 2 Department of Chemistry & Biochemistry, Texas Christian University, Fort Worth, TX 76129, USA; a.encisobarros@tcu.edu (A.E.E.); mirza.hasan@tcu.edu (M.H.); v.costa@tcu.edu (V.C.P.C.); o.annunziata@tcu.edu (O.A.); j.coffer@tcu.edu (J.L.C.)

**Keywords:** dendrimer, triazine, LLPS, LCST, thermoresponsive, nanoparticle, gold

## Abstract

The synthesis and solubility behaviors of four generation five (G5) triazine dendrimers are studied. While the underivatized cationic dendrimer is soluble in water, the acetylated and propanoylated derivatives undergo coacervation in water upon increasing temperature. Occurring around room temperature, this behavior is related to a liquid-liquid phase transition with a lower critical solution temperature (LCST) and is explained by differences in composition, notably, the hydrophobic nature of the terminal groups. Interestingly, the water solubility of the acetylated dendrimer is affected by the addition of selected metal ions. Titrating solutions of acetylated dendrimer at temperatures below the LCST with gold or palladium ions promoted precipitation, but platinum, iridium, and copper did not. Gold nanoparticles having diameters of 2.5 ± 0.8 nm can be obtained from solutions of the acetylated dendrimer at concentrations of gold less than that required to induce precipitation by treating the solution with sodium borohydride.

## 1. Introduction

The temperature-induced, reversible coacervation of macromolecules in solution is a result of liquid-liquid phase separation (LLPS) [1]. Depending on the chemical nature of macromolecule and solvent, LLPS may be induced either by decreasing or increasing temperature. The corresponding temperature-composition phase diagrams will exhibit either an upper critical solution temperature (UCST) or a lower critical solution temperature (LCST), respectively.

This phase transition has been extensively investigated for solutions of linear polymers due to their importance for mixture thermodynamics, separation technologies, self-assembly processes, catalysis and the preparation of thermoresponsive materials [1,2,3,4,5]. However, corresponding studies on dendrimer solutions are scarce [6], which is surprising given the significant overlap between the scopes of dendrimers and LLPS applications. In the case of catalysis, a thermoregulated formation of coacervates of dendrimers could be employed to separate these nanoreactors from the reaction products. In the case of extraction, LLPS could be used to separate the molecules sequestered by the host dendrimers from solution with applications to purification and drug loading. Finally, the coupling of coacervation with chemical crosslinking could be applied to produce crosslinked coacervates with high guest loading capacity, relevant to drug delivery applications.

Our own interest rests in triazine dendrimers [7]. Recent interest in metal nanoparticles led to the serendipitous discovery of dendrimers displaying LLPS behaviors. Specifically, two of these systems undergo reversible opacification upon increasing temperature, characteristic of the LCST type of behavior. To our knowledge, no previous LLPS study has been reported for solutions of triazine dendrimers.

## 2. Results and Discussion

### 2.1. Design and Synthesis

**Figure 1:**
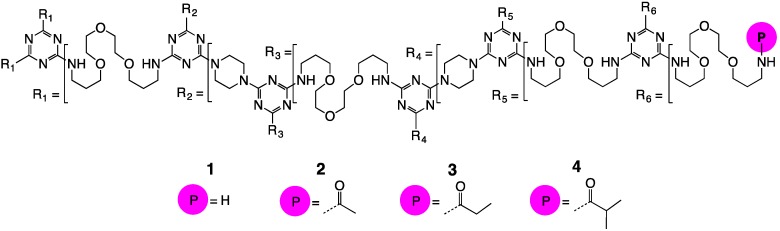
The molecules used in this study.

The compounds examined in this study derive from **1** (Figure 1), a generation 5 triazine dendrimer composed of triazines linked by 4,7,10-trioxodecanediamine and piperazine [8,9,10]. Like **1**, dendrimers **2**, **3**, and **4** are generation 5 dendrimers with 96 end groups and molecular weights of approximately 40 kDa. The compound numbers (**2**, **3**, **4**) conveniently reflect the number of carbons in their acyl groups (acetyl, propanoyl, isobutanoyl).

Generation 5 dendrimers were chosen because this generation shows an onset of globular structure with high degrees of porosity and diameters measuring 6 nm [10]. This size was considered ideal for the creation of dendrimer-encapsulated nanoparticles, the initial impetus for this work [11,12,13,14,15,16,17,18,19,20,21,22,23,24]. The choice of linkers reflects a balance of needs for reactivity during synthesis (piperazine groups) [25,26] and water solubility (4,7,10-trioxodecanediamine) [8,9,10,27]. Additionally, polyethyleneglycol tethers have been shown to influence Au nanoparticle formation and stability [19,20].

The acetylated dendrimer, **2**, was the first target based on literature precedent. Wang *et al*. have shown that acetylated PAMAM dendrimers lead to the formation of dendrimer-encapsulated nanoparticles that are more biocompatible [22]. Pietsch has shown that acetylation leads to better control over nanoparticle size and shape [23].

**Figure 2:**
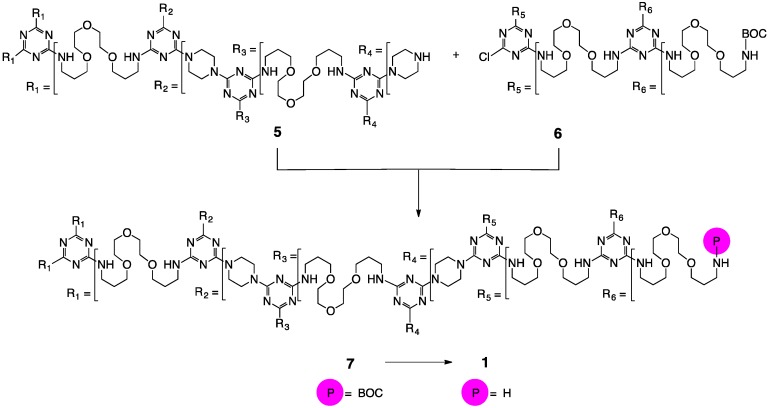
The synthesis of **1** relies on the use of **5** and **6** [12].

To arrive at **2**, progenitor **1** was acetylated with acetic anhydride. Indeed, to arrive at **3** and **4**, **1** was subjected the commercially available acid anhydrides as well. The preparation of **1** relied on condensing two published molecules, **5** and **6** (Figure 2) to yield **7**. Both **5** and **6** are available by a rapid, microwave and macromonomer-mediate synthesis [8,9]. In addition to providing water solubility, these building blocks were perceived to yield dendrimers that were both large and flexible with pores envisioned to support nanoparticle growth. The reaction is facilitated by the presentation of secondary amines (piperazine) on **5** that show higher reactivity than primary amines [25,26] for monochlorotriazines like **6**. Upon isolation of **7**, a BOC-derivative displaying no water solubility, **1** can be obtained by treatment with acid.

During the course of manipulating **2**, we observed an LCST in water. Manipulating the LCST of a system is commonly done by affecting the hydrophobic/hydrophilic balance. In polyacrylamides, for example, the LCST of a polymer comprising *N*-isopropyl groups can be increased by introducing *N*-ethyl groups or decreased by introducing isobutyl groups [28]. To probe the impact that the acyl group has on LCST, the acetylated dendrimer, **2**, was compared with those presenting propanoyl groups, **3**, and isobutanoyl groups, **4**. Like **1**, **4** was insoluble in water at room temperature, precluding further analysis.

### 2.2. Liquid-Liquid Phase Separaration (LLPS) Temperatures

**Figure 3:**
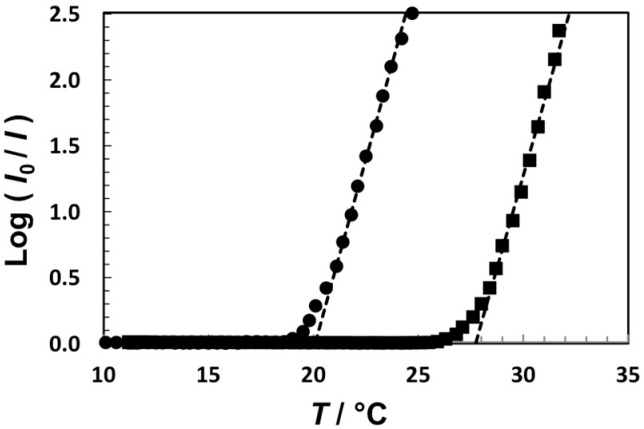
Temperature-turbidity profiles for aqueous solutions of **2** (squares) and **3** (circles). The dendrimer concentration is 4 mg/mL in both cases. The dashes lines, which are linear fits through the data with Log (*I*_0_/*I*) > 0.5, show the values of *T*_ph_ = 20 °C (**3**) and *T*_ph_ = 28 °C (**2**) extrapolated at Log (*I*_0_/*I*) = 0.

LLPS temperatures, *T_ph_*, were determined using the turbidity method described in the Experimental Section. The temperature-induced opacification of aqueous solutions of **2** and **3** at 4 mg/mL are shown in Figure 3. We obtain *T_ph_* = 28 °C for **2** and *T_ph_* = 20 °C for **3**.

LCST behavior is observed for aqueous solutions of polyethylene glycol (PEG) [29]. At room temperature, water is a good solvent for this polymer due to the formation of hydrogen bonds between the PEG ethoxy groups and water molecules. As temperature increases, water becomes a poorer solvent for PEG, thereby leading to LLPS [30]. In our case, PEG-like linkers are used between triazines to enhance water solubility of these dendrimers at room temperature. The observed LCST type of behavior is attributed to the PEG-like domains of our triazine dendrimers. As temperature increases, the hydration of the PEG-like linkers decreases [31], and the solubility of triazine dendrimers is expected to reduce giving rise to LLPS. Similar desolvation behavior of the PEG-like domains has been observed in computational models of these and other dendrimers [32].

The actual location of the LLPS temperature is expected to strongly depend on the chemical nature of the dendrimer terminal groups. That the value of *T_ph_* in the case of **3** is lower than that in the case of **2** correlates with the higher hydrophobicity of the propanoyl group compared to that of the acetyl group. Furthermore, our analysis suggests that the LLPS of aqueous solutions of **4** should be located at lower temperatures, consistent with the observed poor solubility of **4** in water. In summary, our findings show that the modifications of the terminal groups of these triazine dendrimers can be used to modulate the LLPS temperature around room temperature.

### 2.3. Influence of Metal Ions on Solubility of ***2***

Dendrimer **2** was chosen for hosting nanoparticles because the LCST was sufficiently high to facilitate their synthesis. However, upon adding approximately 100 mole equivalents of gold in the form of HAuCl_4_-3H_2_O to solutions of **2**, a precipitate formed. While we recognize that the equivalence point is close to the number of end groups, additional studies will be required to ascertain the mechanistic basis for this coincidence. A similar behavior was seen with additions of Na_2_PdCl_4_. In contrast, titrated additions of K_2_PtCl_4_, IrCl_3_∙xH_2_O, or CuSO_4_∙5H_2_O did not promote precipitation. The selectivity observed could be useful, as the separation of gold or palladium from copper is of some interest in the recycling of microelectronic waste streams [33,34,35,36]. Simple sequestration strategies are also attracting attention [37,38]. Dendrimers are largely unexplored in this capacity, but the scalable synthesis of triazines makes such opportunities of interest.

### 2.4. Formation of Nanoparticles

**Figure 4:**
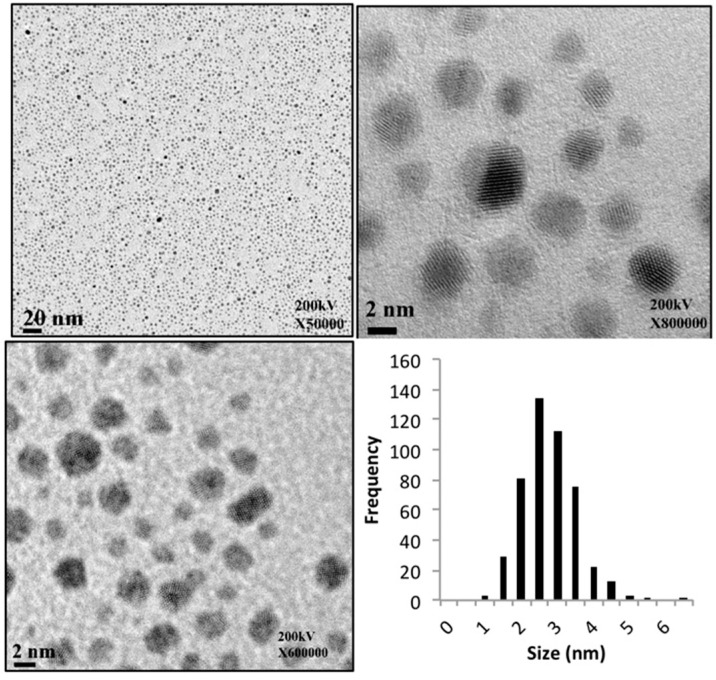
A TEM micrographs at three magnifications of dendrimer-encapsulated gold nanoparticles derived from **2** and the distribution of their sizes.

At low molar ratios of Au^3+^: Dendrimer (approximately 60:1), soluble dendrimer-encapsulated nanoparticles could be prepared by the addition of sodium borohydride, a reagent required to generate Au at room temperature. Figure 4 summarizes the results of these experiments. The mean diameters of these particles was 2.55 ± 0.84 nm. The particles are red in solution and UV-Vis spectroscopy reveals an absorption maximum at 510 nm, consistent with the well-known surface plasmon of gold. Preliminary experiments show that nanoparticles of palladium, platinum, iridium and copper are also accessible, but these studies are preliminary and will be reported in due course. For gold nanoparticles, we note that not only is the particle size reasonably homogeneous, but the spacing between the particles is very similar and close to 6 nm, the diameter of the dendrimer. That is, the micrograph is consistent with a single gold nanoparticle encapsulated within a single globular macromolecule that sterically separates it from its neighbor. Additional experiments will be required to bear out this hypothesis.

## 3. Materials and Methods

### 3.1. General Experimental

All reagents were used as received. Methanol, dichloromethane, dioxane, diethylether, acetic anhydride, HAuCl_4_, D_2_O, CDCl_3_ (Sigma-Aldrich, St. Louis, MO, USA); triethylamine (TEA), DIPEA, Na_2_PdCl_4_, K_2_PtCl_4_, IrCl_3_∙x H_2_O (Pressure Chemical, Pittsburgh, PA, USA). Microwave reactions were carried on using a CEM SP Discover microwave (CEM Corporation, Matthews, NC, USA). NMR experiments were conducted on a Bruker 400 Ascend spectrometer (Bruker, Billerica, MA, USA); UV-Vis spectra were obtained on an Agilent 8453(Agilent, Santa Clara, CA, USA) were recorded at room temperature in water 18 mΩ. The NMR data listed shows the theoretical number of protons expected for the molecule reported. Error prevents an accurate assessment of the true numbers, but integration of the spectra corroborate this expectation.

### 3.2. Preparation of ***1***

A solution of **7** (0.2605 g, 4 µmol) in dioxane (6 mL) was mixed with HCl conc. (3 mL) and heated 3 min at 60 °C using dynamic mode. Afterwards, solvent was evaporated under vacuum and residue dissolved in water; pH was adjusted to 12 using 5 M NaOH (aq). The product was extracted with dichloromethane and, after solvent evaporation, deprotected dendrimer was obtained as a white thick oil (0.2069 g, quantitative yield). ^1^H-NMR (400 MHz, D_2_O) δ 3.77–3.11 (m, 2592H, CH_2_OCH_2_CH_2_OCH_2_CH_2_OCH_2_, C_3_N_3_-NHCH_2_CH_2_CH_2_O, C_3_N_3_-NCH_2_CH_2_N), δ 2.74–2.92 (br, 192H, NH_2_CH_2_), δ 1.86–1.59 (br, 636H, OCH_2_CH_2_CH_2_NH); ^13^C-NMR (100 MHz, CDCl_3_) δ 166.06 (C_3_N_3_), δ 165.14 (C_3_N_3_), δ 70.57 (OCH_2_CH_2_O), δ 70.16 (two lines, OCH_2_CH_2_O), δ 69.42 (NHCH_2_CH_2_CH_2_O), δ 69.28 (NHCH_2_CH_2_CH_2_O), δ 43.01 (NCH_2_CH_2_N), δ 39.50 (NHCH_2_CH_2_CH_2_O), δ 38.08 (NHCH_2_CH_2_CH_2_O), δ 33.12 (NHCH_2_CH_2_CH_2_O), δ 29.68 (NHCH_2_CH_2_CH_2_O); MS (MALDI-TOF) calcd for C_1992_H_3834_N_660_O_477_ 44639.60, found 40742.54. See the Supplementary Materials (Figures S4–S6) for the NMR and MS data of **1**.

### 3.3. Preparation of ***2***

A mixture of **1** (0.0917 g, 2 µmol), acetic anhydride (93 µL, 985 µmol) and triethylamine (165 µL, 1183 µmol) in methanol (13 mL) was stirred overnight at room temperature. Afterwards, solvent was evaporated and residue dissolved in water; then, impurities were filtered off using ultracentrifugation (30 min, 14,000 rpm) and product was washed two more times with pure water. After liophylization, a pale yellow sticky solid was obtained (0.0497g, 50% yield). ^1^H-NMR (400 MHz, D_2_O) δ 3.12–3.60 (m, 2592H, CH_2_OCH_2_CH_2_OCH_2_CH_2_OCH_2_, C_3_N_3_-NHCH_2_CH_2_CH_2_O, C_3_N_3_-NCH_2_CH_2_N), δ 3.05–3.12 (t, 192H AcNHCH_2_), δ 1.57–1.77 (br, 636H, OCH_2_CH_2_CH_2_NH), δ 1.84 (s, 288H, COCH_3_; ^13^C-NMR (100 MHz, D_2_O) δ 173.44 (CO), δ 164.76 (br, C_3_N_3_), δ 69.70 (OCH_2_CH_2_O), δ 69.42 (two lines, OCH_2_CH_2_O), δ 68.64 (NHCH_2_CH_2_CH_2_O), δ 68.45 (NHCH_2_CH_2_CH_2_O), δ 42.88 (NCH_2_CH_2_N), δ 37.50 (NHCH_2_CH_2_CH_2_O), δ 36.49 (NHCH_2_CH_2_CH_2_O), δ 28.96 (NHCH_2_CH_2_CH_2_O), δ 28.35 (NHCH_2_CH_2_CH_2_O), δ 21.89 (C(CO)); MS (MALDI-TOF) calcd for C_2184_H_4026_N_660_O_573_ 48672.62, found 40652.96. See the Supplementary Materials (Figures S7–S9) for the NMR and MS data of **2**.

### 3.4. Preparation of ***3***

A mixture of **1** (0.039 g, 0.873 µmol), propionic anhydride (53 µL, 419 µmol) and triethylamine (70 µL, 503 µmol) in methanol (10 mL) was stirred overnight at room temperature. Afterwards, solvent was evaporated and residue dissolved in water; then, impurities were filtered off using ultracentrifugation (30 min, 14,000 rpm) and product was washed two more times with pure water. After liophylization, a pale yellow sticky solid was obtained (0.009 g, 21% yield). ^1^H-NMR (400 MHz, D_2_O) δ 3.10–3.60 (m, 2592H, CH_2_OCH_2_CH_2_OCH_2_CH_2_OCH_2_, C_3_N_3_-NHCH_2_CH_2_CH_2_O, C_3_N_3_-NCH_2_CH_2_N), δ 2.10–2.04 (m, 192H CH_3_CH_2_CONHCH_2_), δ 1.45–1.95 (br, 636H, OCH_2_CH_2_CH_2_NH), δ 0.91–0.97 (t, 288H, COCH_2_CH_3_); ^13^C-NMR (100 MHz, D_2_O) δ 176.6 (CO),δ 165.2 (br, C_3_N_3_), δ 69.7 (OCH_2_CH_2_O), δ 69.4 (two lines, OCH_2_CH_2_O), δ 68.5 (NHCH_2_CH_2_CH_2_O), δ 42.88 (NCH_2_CH_2_N), δ 37.4 (NHCH_2_CH_2_CH_2_O), δ 36.4 (NHCH_2_CH_2_CH_2_O), δ 29.1 (NHCH_2_CH_2_CH_2_O), δ 29.0 (NHCH_2_CH_2_CH_2_O), δ 28.4 (CH_3_CH_2_CON), δ 9.7 (CH_3_CH_2_CON); MS (MALDI-TOF) calcd for C_2280_H_4218_N_660_O_573_ 50018.12, found 47376.9. See the Supplementary Materials (Figures S10–S12) for the NMR and MS data of **3**.

### 3.5. Preparation of ***4***

A mixture of **1** (0.039 g, 0.873 µmol), isobutyric anhydride (100 µL, 603 µmol) and triethylamine (14 µL, 100µmol) in methanol (10 mL) was heated overnight at 40 °C Afterwards, solvent was evaporated and residue dispersed in water; then, impurities were filtered off using ultracentrifugation (30 min, 14,000 rpm) and product was washed two more times with pure water. After lyophylization, a pale yellow sticky solid was obtained (0.029g, 65% yield). ^1^H-NMR (400 MHz, D_2_O) δ 3.21–3.60 (m, 2592H, CH_2_OCH_2_CH_2_OCH_2_CH_2_OCH_2_, C_3_N_3_-NHCH_2_CH_2_CH_2_O, C_3_N_3_-NCH_2_CH_2_N), δ 3.05–3.22 (m, 192H CH_3_CH_2_CONHCH_2_), δ 2.31–2.38 (m, 96, CH(CH_3_)_2_) , δ 1.64–1.73 (br, 636H, OCH_2_CH_2_CH_2_NH), δ 0.99–0.97 (d, 576H, CH(CH_3_)_2_); ^13^C-NMR (100 MHz, D_2_O) δ 180.0 (CO),δ 165.2 (br, C_3_N_3_), δ 69.7 (OCH_2_CH_2_O), δ 69.5 (OCH_2_CH_2_O), δ 69.4 (OCH_2_CH_2_O), δ 68.5 (NHCH_2_CH_2_CH_2_O), δ 42.88 (NCH_2_CH_2_N), δ 38.2 (NHCH_2_CH_2_CH_2_O), δ 37.6 (NHCH_2_CH_2_CH_2_O), δ 35.1 (CH(CH_3_)_2_), δ 28.7 (NHCH_2_CH_2_CH_2_O), δ 18.7 (CH(CH_3_)_2_); MS (MALDI-TOF) calcd for C_2376_H_4506_N_660_O_573_ 51460.37, not found. See the Supplementary Materials (Figures S13 and S14) for the NMR spectra of **4**.

### 3.6. Preparation of ***7***

Synthesis of **7** was accomplished following an analogous procedure to the one reported for Enciso *et al* [9]. A solution of 5 (0.0711g, 9 µmol) in methanol (0.5 mL) was mixed with another solution of 6 (0.9157 g, 461 µmol) in dioxane (4 mL), and DIPEA (0.2 mL, 1148 µmol) was added. Then mixture was heated for 6 h at 95 °C. Solvent was evaporated and product impurities were removed washing the crude several times with diethyl ether. A white solid, **7**, was obtained (0.3681 g, 71% yield). ^1^H-NMR (400 MHz, CDCl_3_) δ 3.25–3.95 (m, 2592H, CH_2_OCH_2_CH_2_OCH_2_CH_2_OCH_2_, C_3_N_3_-NHCH_2_CH_2_CH_2_O, C_3_N_3_-NCH_2_CH_2_N), δ 3.02–3.25 (br, 192H BocNHCH_2_), δ 1.54–1.96 (br, 636H, OCH_2_CH_2_CH_2_NH), δ 1.38 (s, 864H, C(CH_3_)_3_); ^13^C-NMR (100 MHz, CDCl_3_) δ 164.56 (br, C_3_N_3_), δ 156.04 (CO), δ 78.77 (C(CH_3_)_3_), δ 70.53 (OCH_2_CH_2_O), δ 70.15 (two lines, OCH_2_CH_2_O), δ 69.48 (NHCH_2_CH_2_CH_2_O), δ 69.15 (NHCH_2_CH_2_CH_2_O), δ 43.0 (NCH_2_CH_2_N), δ 38.24 (NHCH_2_CH_2_CH_2_O), δ 38.45(NHCH_2_CH_2_CH_2_O), δ 29.58 (NHCH_2_CH_2_CH_2_O), δ 29.46 (NHCH_2_CH_2_CH_2_O), δ 28.44 (C(CH_3_)_3_); MS (MALDI-TOF) calcd for C_2472_H_4602_N_660_O_669_ 54244.64, found 50180.07. See the Supplementary Materials (Figures S1–S3) for the NMR and MS data of **7**.

### 3.7. Measurement of the Cloud Point

The phase separation temperature, *T_ph_*, was determined by measuring the turbidity of binary dendrimer-water samples as function of temperature. A binary dendrimer-water homogenous sample with a dendrimer concentration of 4 mg/mL was prepared by mixing known amounts of water and dendrimer. The turbidity meter is comprised of a programmable circulating bath (1197P, VWR), a calibrated thermocouple (±0.1 °C), a homemade optical cell, where the initially-transparent sample (optical path of 0.4 cm) and a thermocouple probe are located. Collimated light from a solid state laser (633 nm, 5 mW, Coherent) passes through the sample and its transmittance is recorded by a photodiode detector coupled with a computer-interfaced optical meter (1835-C Newport). After recording the transmitted intensity of the transparent sample, *I_o_*, the temperature of the bath is changed at a constant rate of ±0.5 °C/min and the transmitted intensity, *I*, is recorded as a function of temperature, *T.* We identify *T_ph_* as the temperature at which a sharp decrease in intensity is observed (see Figure 3).

### 3.8. Titrations

A stock solution of **2** (0.0184 g, 0.37 µmol) in water (3 mL) were prepared. For every complexation experiment, 100 µL of stock solution were diluted with 900 µL of water in a 1 mL quartz cuvette to yield a dendrimer concentration (1.25 × 10^−5^ M). Aqueous solutions of metal salts (typically at 10 × lower concentration) were freshly prepared and added under stirring using a micropipette; no more than 100 µL were added to the dendrimer solution to avoid dilution effects; mixtures were allowed to equilibrate 10 minutes (Cu, Pt, Ir, and Au) or 15 min (Pd).

### 3.9. Nanoparticle Synthesis

The same stock solution of metal-dendrimer (**2**) titration experiments was used. For every reduction experiment 100 µL of stock solution were diluted with 900 µL of water in a 1 mL quartz cuvette. Aqueous solutions of metal precursors were freshly prepared and added under stirring using a micropipette (30 µL, 60 metal equivalents). Mixture was left to equilibrate for a period of 15 min before NaBH_4_ were added (20 µL, metal to NaBH_4_ ratio 1:4) under vigorous stirring. The procedure was carried out at room temperature.

### 3.10. Electron Microscopy

Samples of dendrimer-encapsulated gold nanoparticles were analyzed using transmission electron microscopy (JEOL Ltd., Tokyo, Japan) on a JEOL JEM-2100 operating at 200 kV. Approximately 50 μL of a given sample was applied to a carbon-coated copper grid and allowed to air dry.

## 4. Conclusions

Aqueous solutions of some generation 5 triazine dendrimers display LLPS with an LCST type of behavior. The LCST of these materials can be varied with the choice of terminal acyl group: Acetylated dendrimers remain soluble at higher temperatures than propanoylated dendrimers. Curiously, the LCST appears to be affected by the addition of some metal ions, but not others. Indeed, this behavior could translate into strategies for the selective recovery of some precious metal salts like gold and palladium in the presence of less valuable ones such as copper. Gold nanoparticles can still be achieved, however, by performing the reduction at concentrations below which precipitation occurs.

## Supplementary Materials

Supplementary materials can be accessed at: http://www.mdpi.com/1420-3049/21/5/599/s1.

## Abbreviations

The following abbreviations are used in this manuscript:

DEN: Dendrimer-encapsulated nanoparticle
G5: Generation 5
LCST: Lower critical solution temperature
LLPS: Liquid-liquid phase separation
PAMAM: Polyamidoamine
PPI: Polypropyleneimine
NaBH_4_: Sodium tetrahydroborane
TEM: Transmission electron microscopy
