# Supplementary material for: Thermoregulated Coacervation, Metal-Encapsulation and Nanoparticle Synthesis in Novel Triazine Dendrimers
Source: Molecules. 2016 May 11;21(5):599. doi: 10.3390/molecules21050599 (PMC6273988; doi:10.3390/molecules21050599)
Supplement: Supplementary file 1 [file molecules-21-00599-s001.pdf]

Supplementary Materials: Thermoregulated Coacervation, Metal-Encapsulation and Nanoparticle Synthesis in Novel Triazine Dendrimers

Fermín Ramírez-Crescencio, Alan E. Enciso, Mirza Hasan, Viviana C. P. da Costa, Onofrio Annunziata, Rocío Redón, Jeffrey L. Coffer and Eric E. Simanek

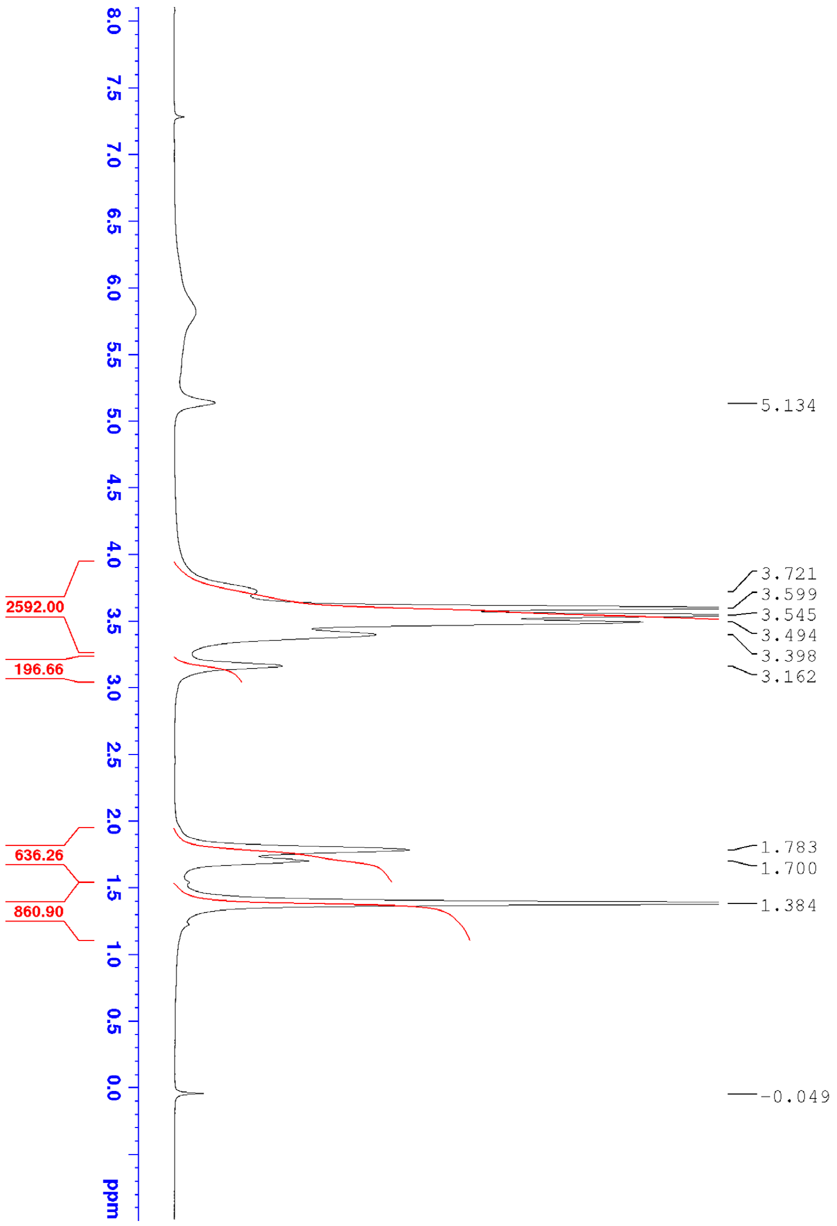

Figure S1. ^{1}H-NMR Spectrum of **7** (400 MHz, $CDCl_3$).

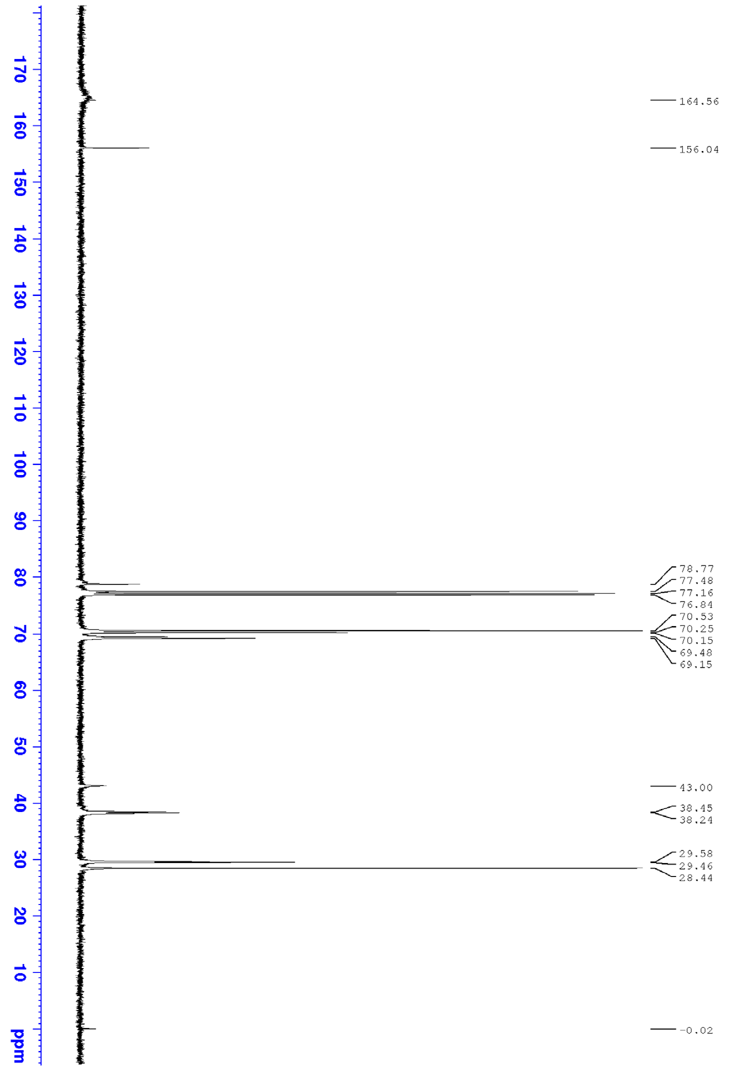

Figure S2. ^{13}C-NMR Spectrum of **7** (100 MHz, $CDCl_3$).

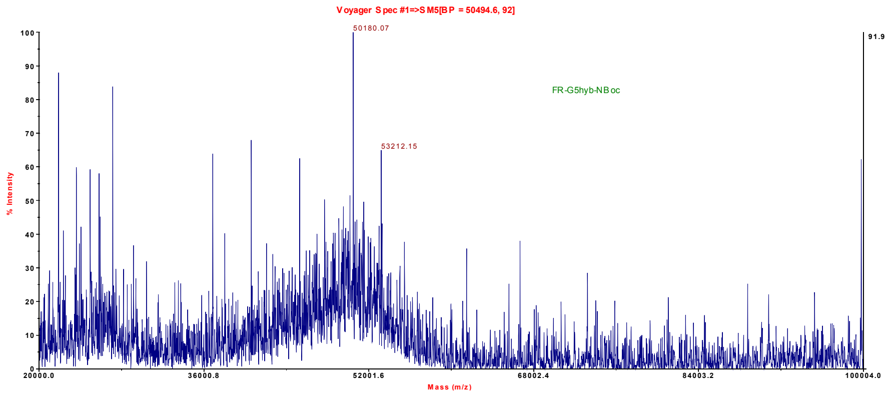

Figure S3. MALDI-TOF analysis of **7** in THAP matrix.

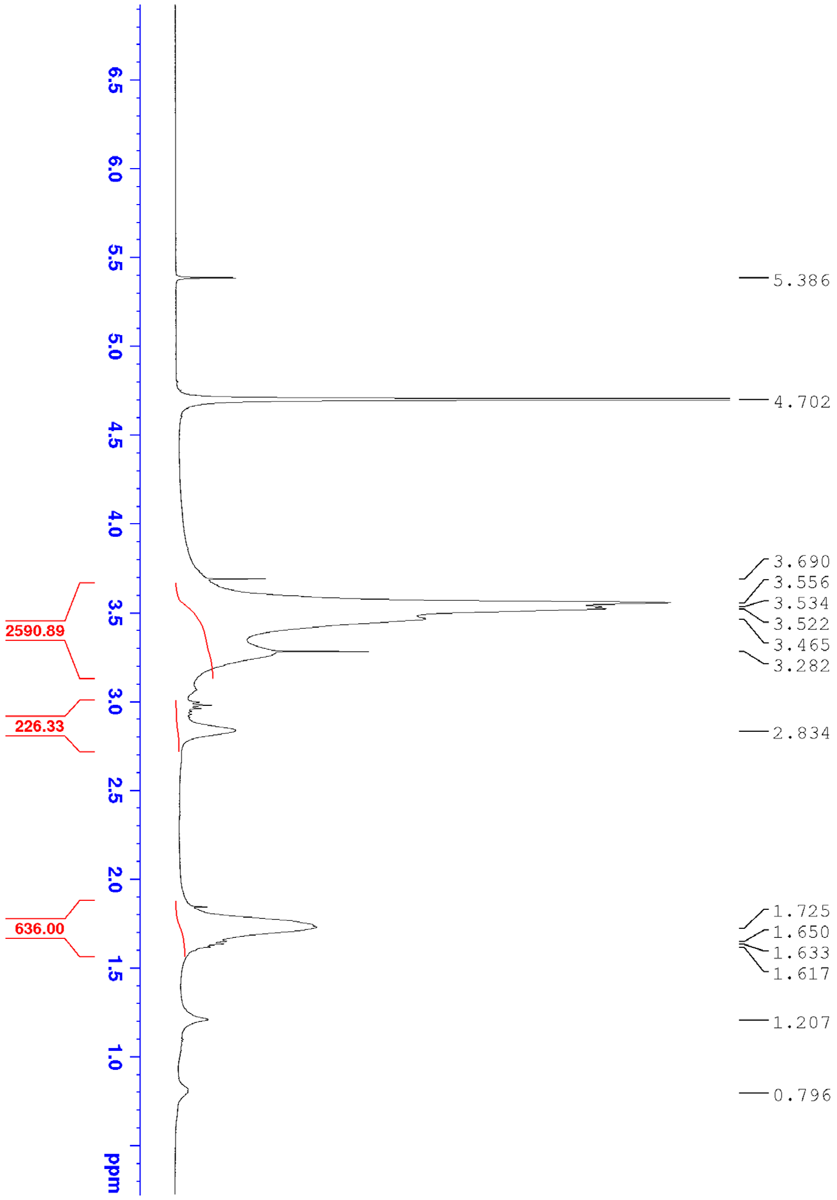

Figure S4. ^{1}H-NMR Spectrum of **1** (400 MHz, D_2O).

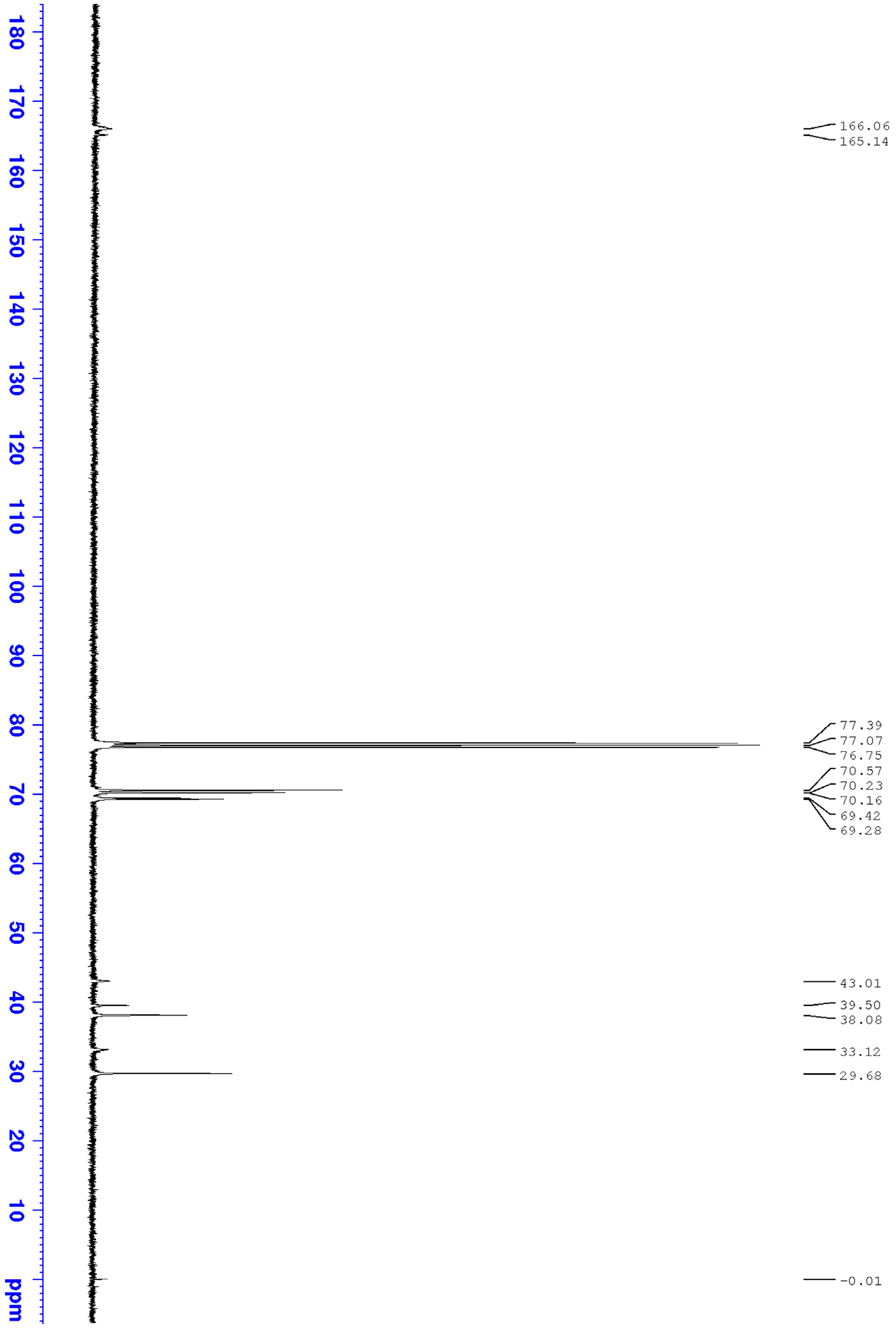

Figure S5. ^{13}C-NMR Spectrum of **1** (100 MHz, D_2O).

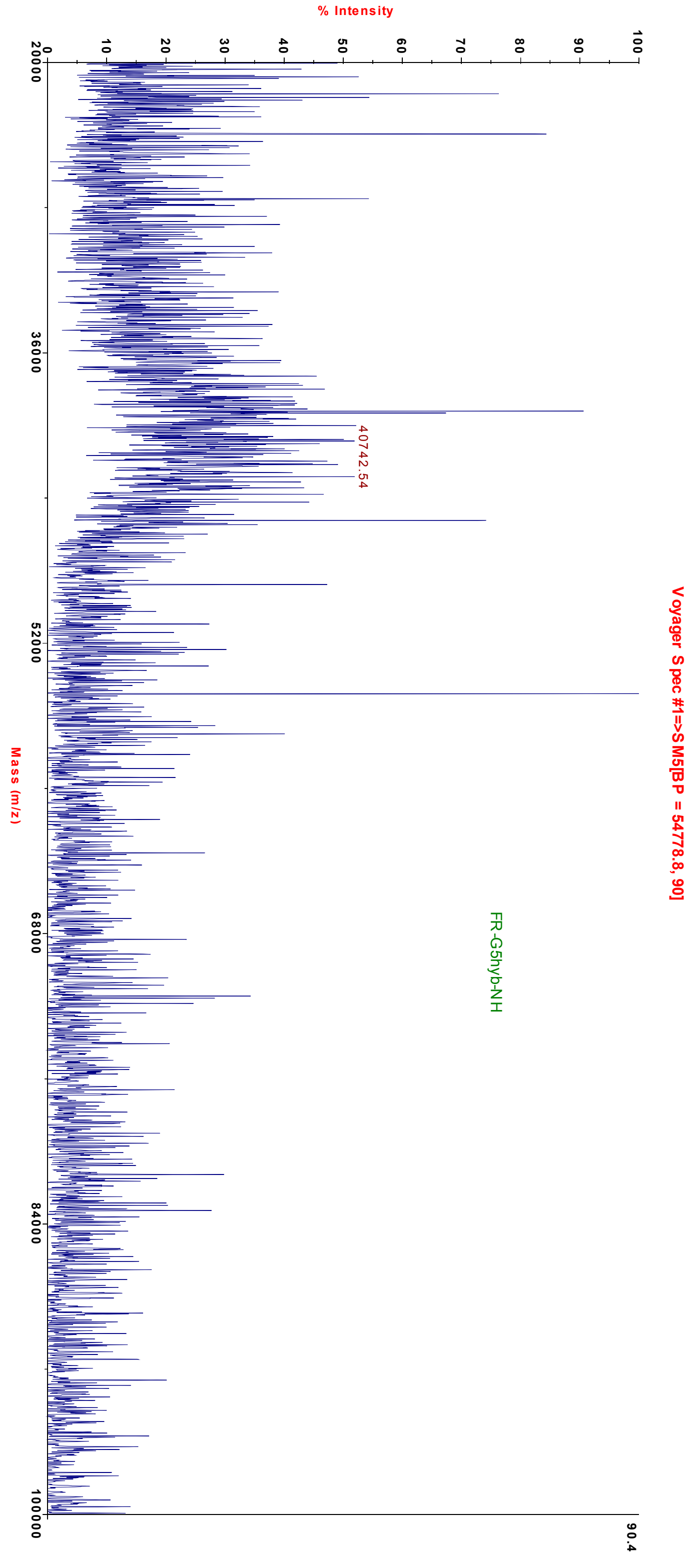

Figure S6. MALDI-TOF analysis of **1** in THAP matrix.

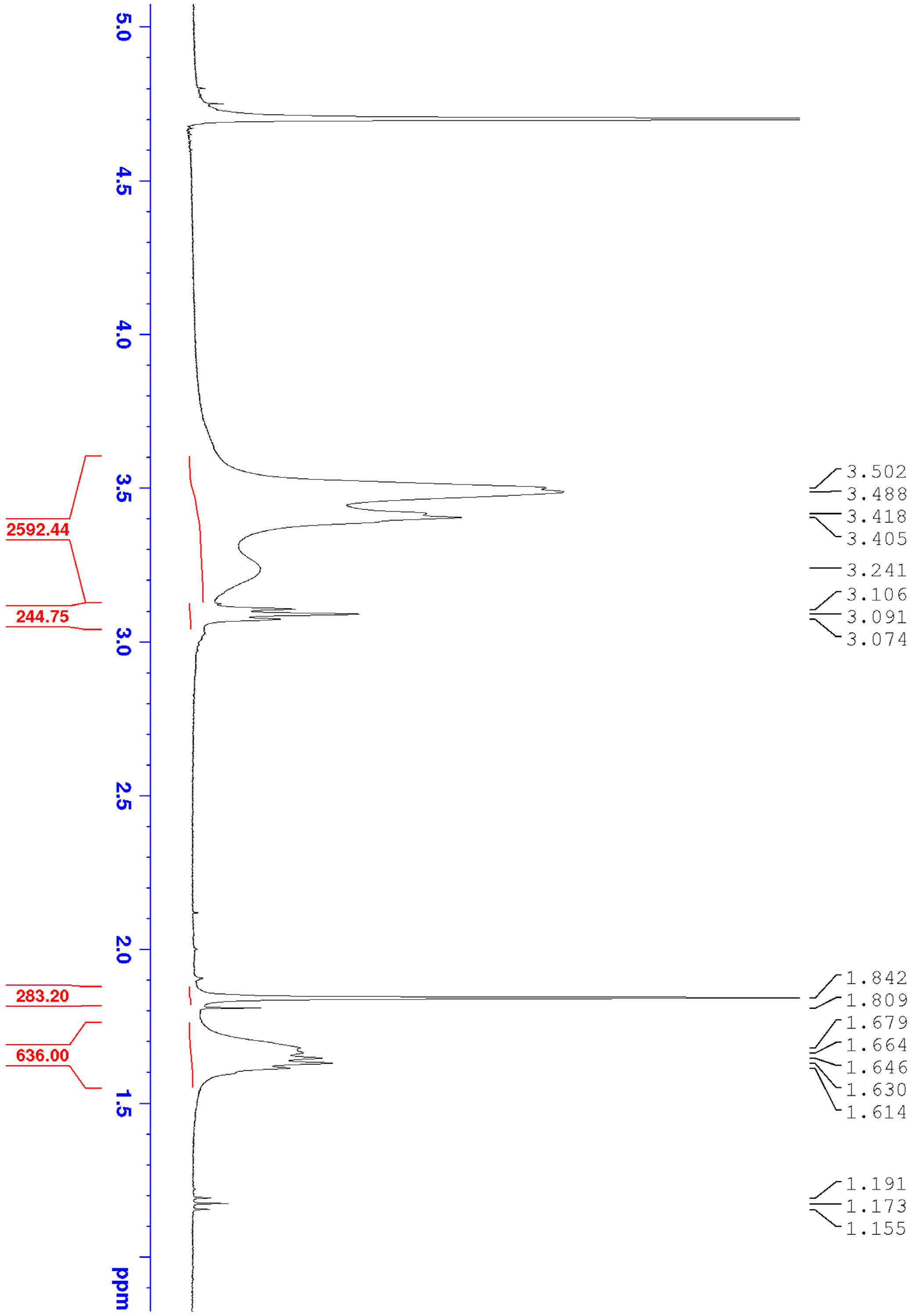

Figure S7. ^{1}H-NMR Spectrum of **2** (400 MHz, D_2O).

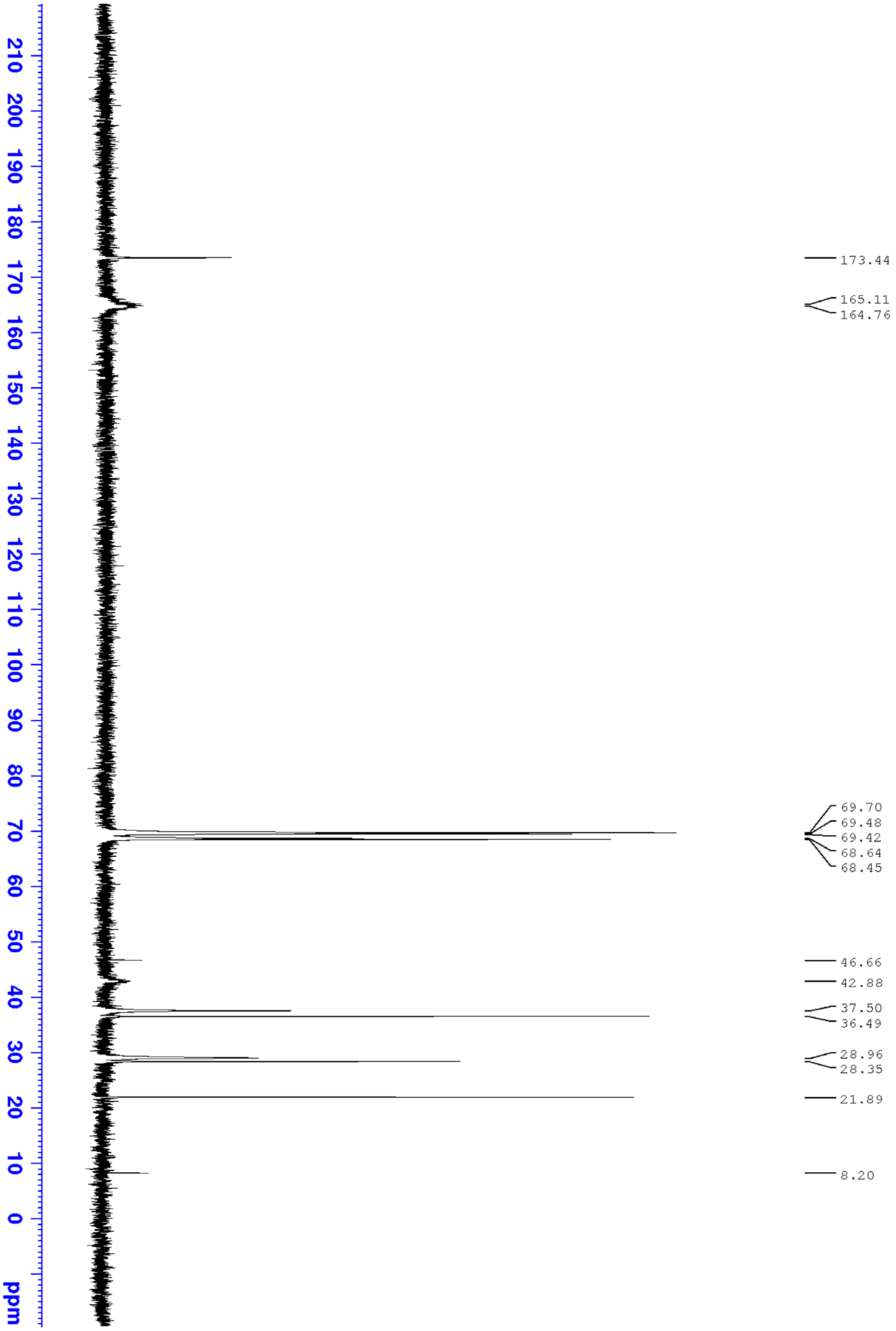

Figure S8. ^{13}C-NMR Spectrum of **2** (100 MHz, D_2O).

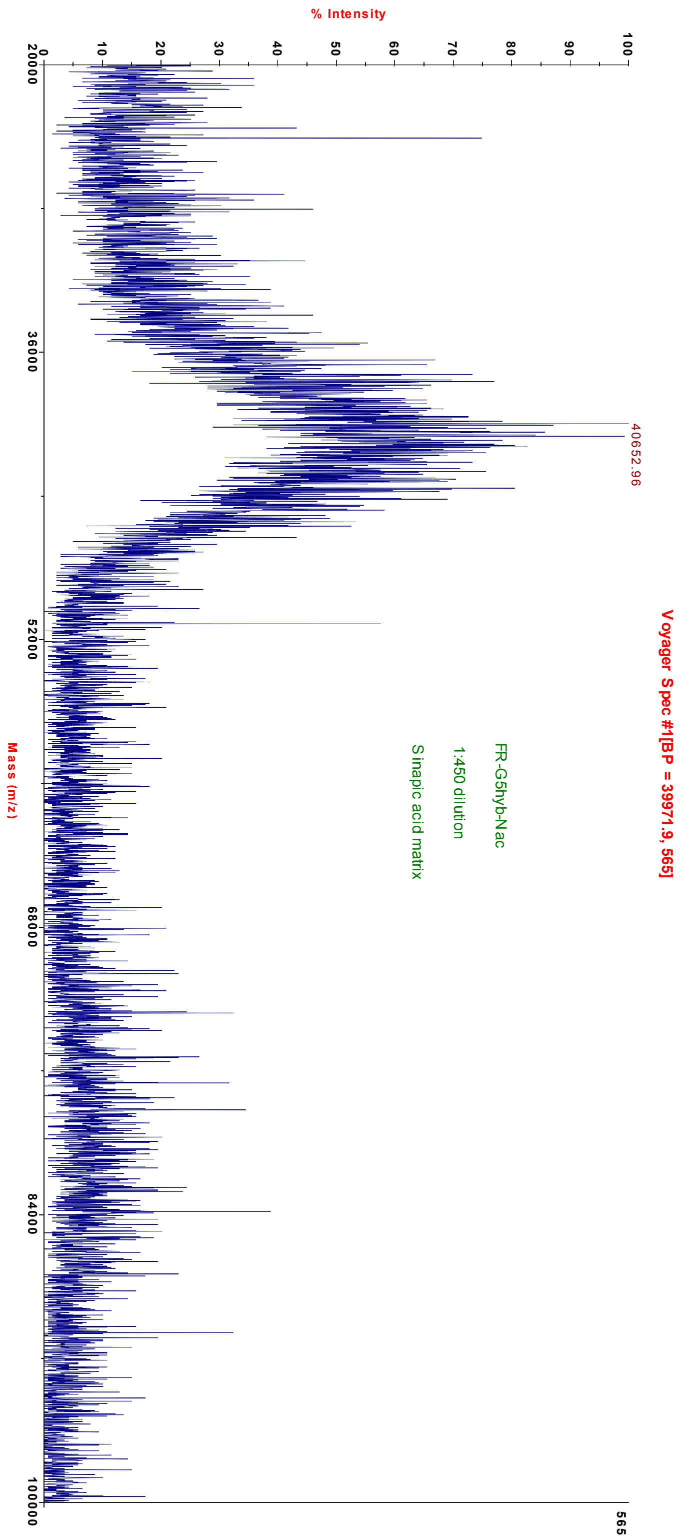

Figure S9. MALDI-TOF analysis of **2** in sinapic acid matrix.

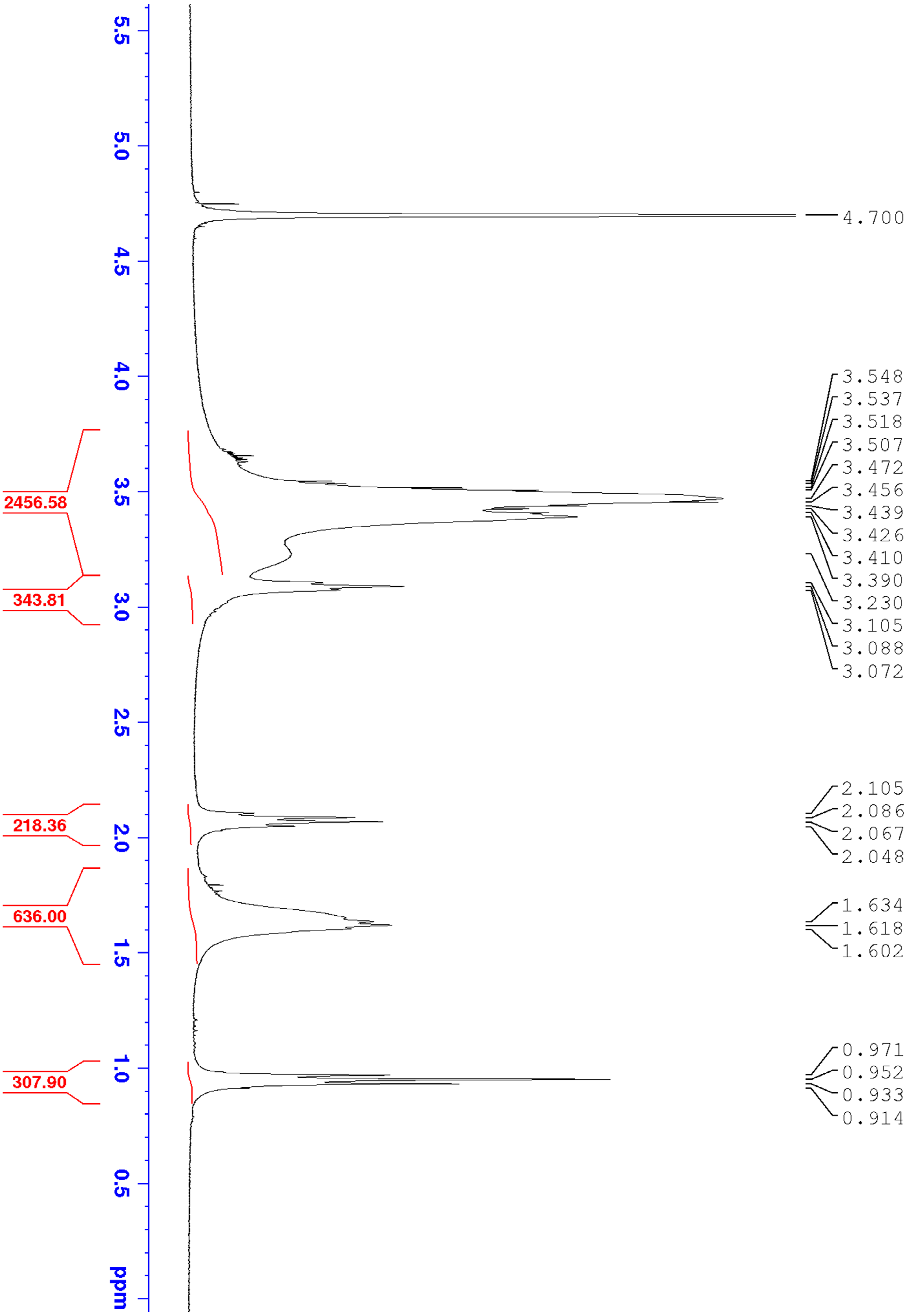

Figure S10. ^{1}H-NMR Spectrum of **3** (400 MHz, D_2O).

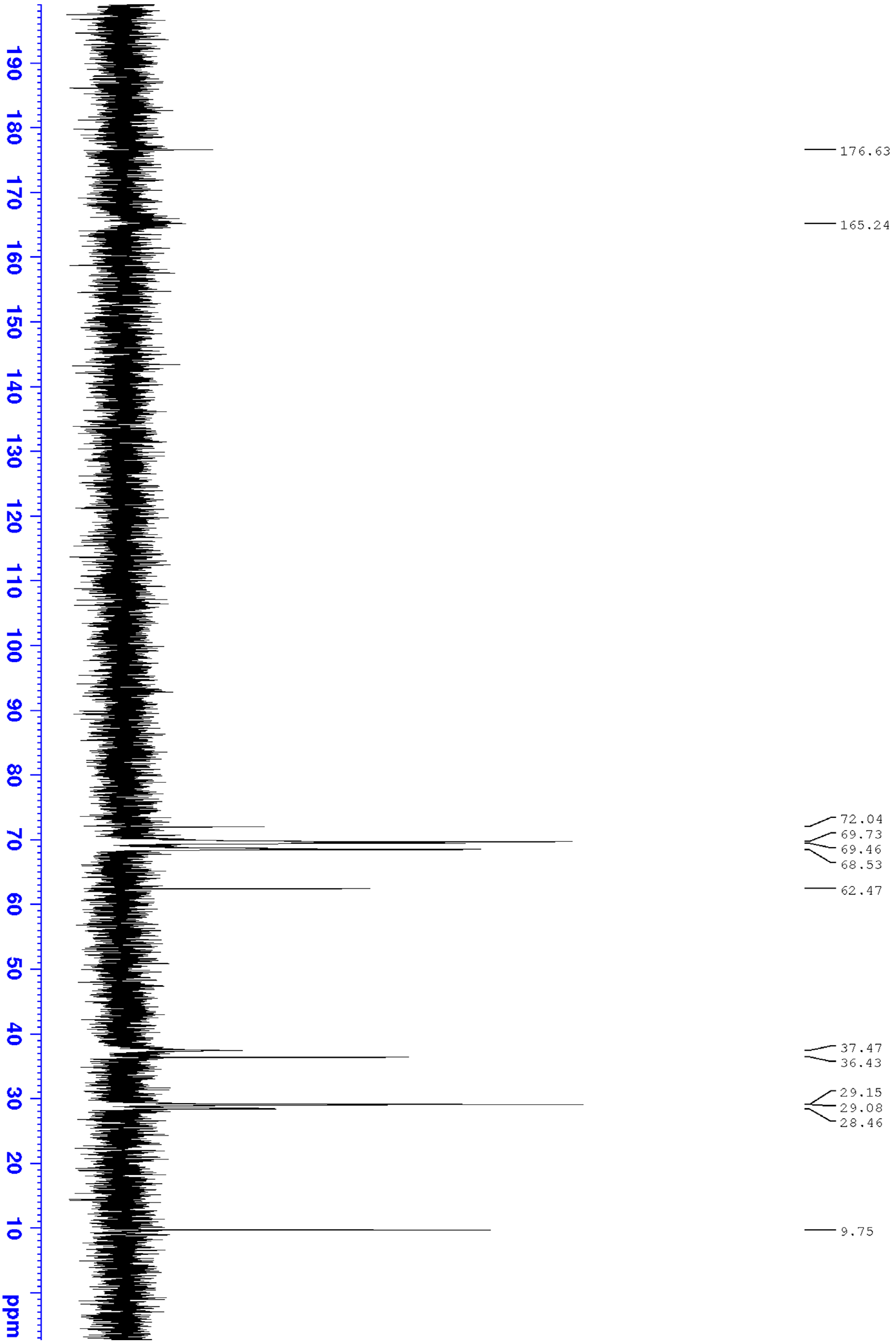

Figure S11. ^{13}C-NMR Spectrum of **3** (100 MHz, D_2O).

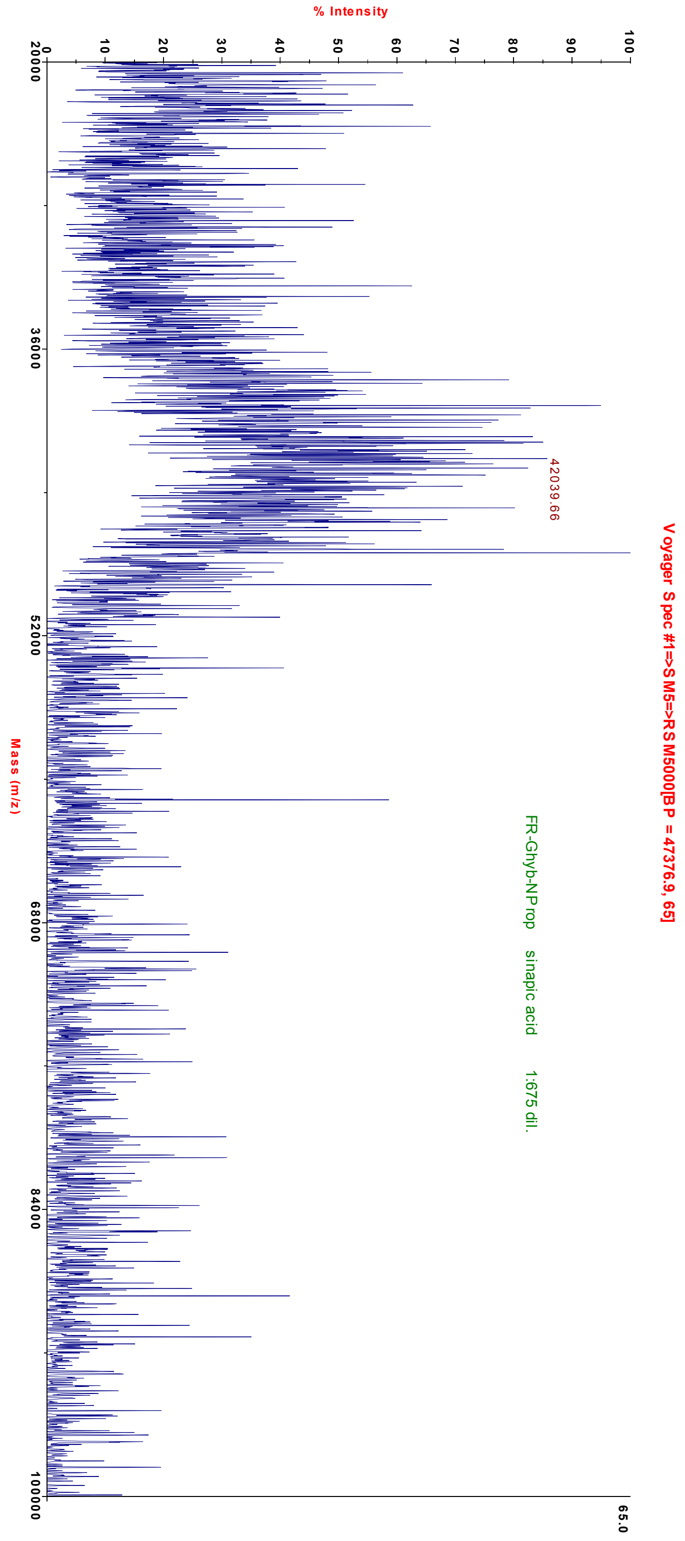

Figure S12. MALDI-TOF analysis of **3** in sinapic acid matrix.

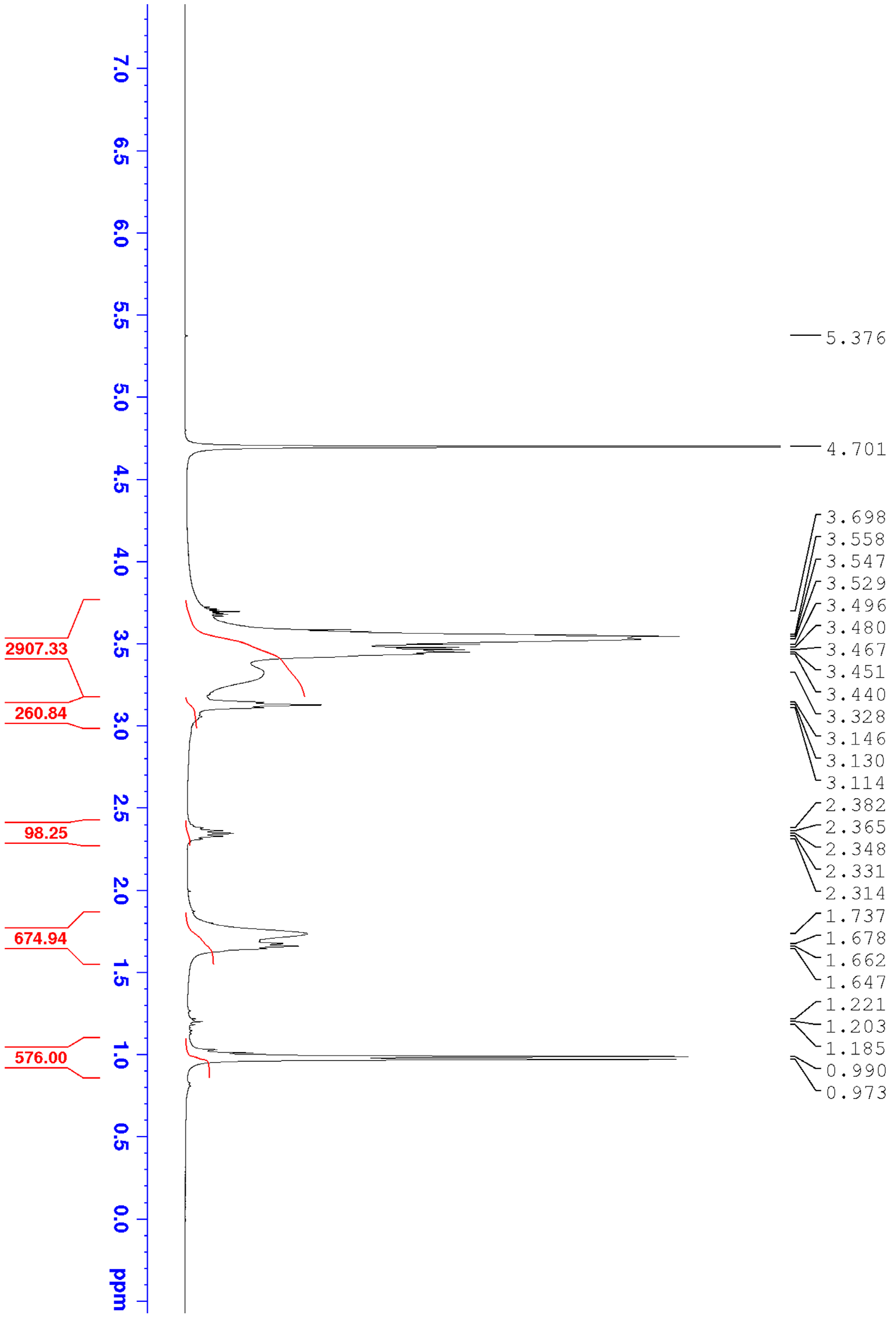

Figure S13. ^{1}H-NMR Spectrum of **4** (400 MHz, D_2O).

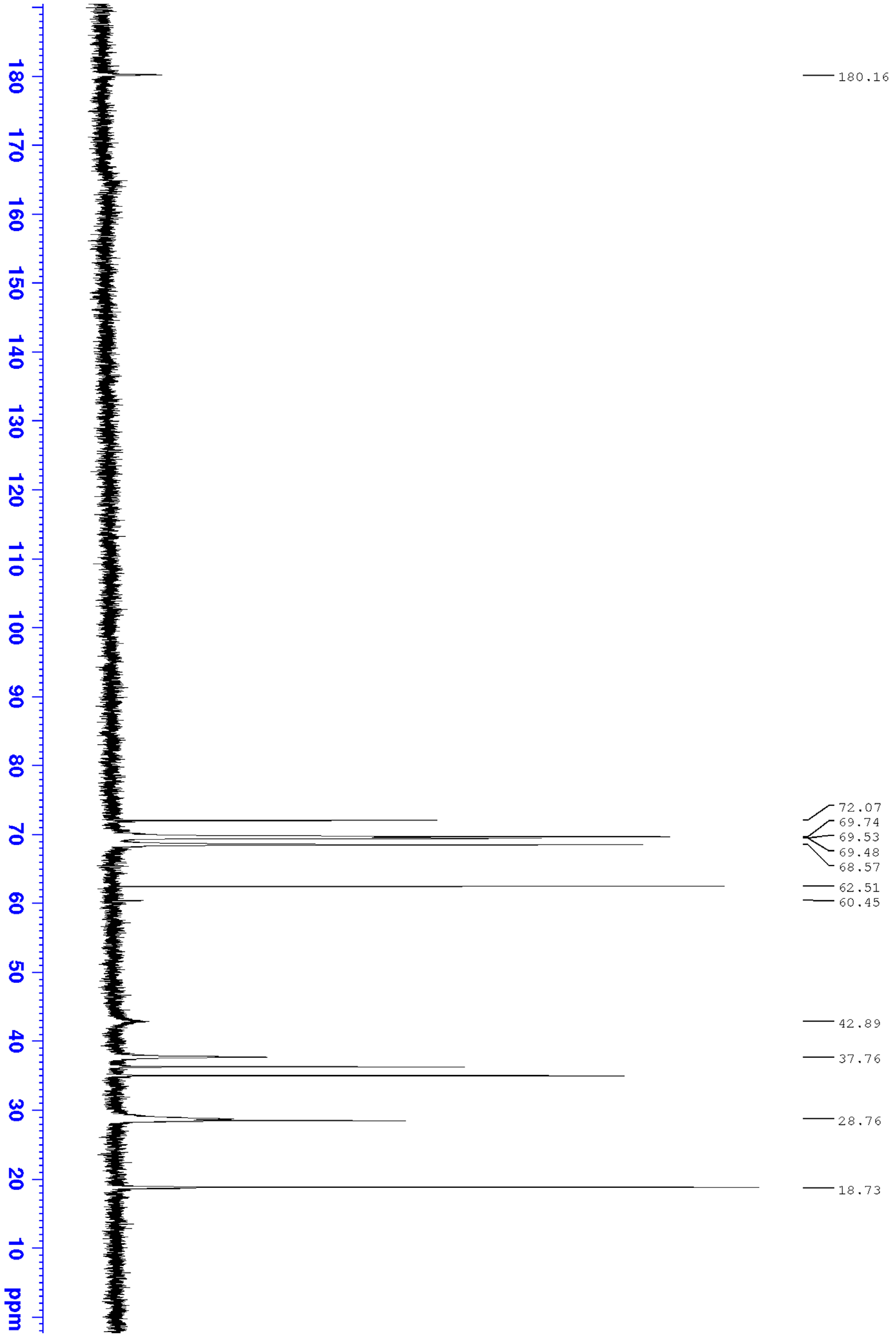

Figure S14. ^{13}C-NMR Spectrum of **4** (100 MHz, D_2O).
